# Phase II Study of the Liposomal Formulation of Eribulin (E7389-LF) in Combination with Nivolumab: Results from the Small Cell Lung Cancer Cohort

**DOI:** 10.1158/2767-9764.CRC-23-0313

**Published:** 2024-01-29

**Authors:** Makoto Nishio, Shuji Murakami, Hisato Kawakami, Kyoichi Okishio, Motohiro Tamiya, Haruki Kobayashi, Daichi Fujimoto, Shunichi Sugawara, Toshiyuki Kozuki, Yuko Oya, Hiroki Izumi, Takayuki Shiroyama, Miyako Satouchi, Noboru Yamamoto, Shota Kaname, Daiko Matsuoka, Yohei Otake, Takao Takase, Taro Semba, Koichi Azuma

**Affiliations:** 1 Department of Thoracic Medical Oncology, Cancer Institute Hospital of the Japanese Foundation for Cancer Research, Tokyo, Japan.; 2 Department of Thoracic Oncology, Kanagawa Cancer Center, Yokohama, Japan.; 3 Medical Oncology, Kindai University Faculty of Medicine, Osakasayama, Japan.; 4 Department of Thoracic Oncology, National Hospital Organization Kinki-Chuo Chest Medical Center, Osaka, Japan.; 5 Department of Thoracic Oncology, Osaka International Cancer Institute, Osaka, Japan.; 6 Division of Thoracic Oncology, Shizuoka Cancer Center, Shizuoka, Japan.; 7 Internal Medicine III, Wakayama Medical University Hospital, Wakayama, Japan.; 8 Pulmonary Medicine, Sendai Kousei Hospital, Sendai, Japan.; 9 Department of Thoracic Oncology and Medicine, National Hospital Organization Shikoku Cancer Center, Matsuyama, Japan.; 10 Department of Respiratory Medicine, Fujita Health University Hospital, Toyoake, Japan. Previous Affiliation: Aichi Cancer Center, Nagoya, Japan.; 11 Department of Thoracic Oncology, National Cancer Center Hospital East, Kashiwa, Japan.; 12 Department of Respiratory Medicine and Clinical Immunology, Osaka University Graduate School of Medicine, Osaka, Japan.; 13 Thoracic Oncology, Hyogo Cancer Center, Hyogo, Japan.; 14 Department of Experimental Therapeutics, National Cancer Center Hospital, Tokyo, Japan.; 15 Oncology Early Clinical Operation II, Ono Pharmaceutical Co., Ltd., Osaka, Japan.; 16 Japan and Asia Clinical Development Department, Oncology, Clinical Evidence Generation Fulfillment, Deep Human Biology Learning, Eisai Co., Ltd., Tokyo, Japan.; 17 Clinical Data Science Department, Clinical Evidence Generation Fulfillment, Deep Human Biology Learning, Eisai Co., Ltd., Tokyo, Japan.; 18 Molecular Profiling Department, Discovery Concept Validation function, Deep Human Biology Learning, Eisai Co., Ltd., Ibaraki, Japan.; 19 Division of Respirology, Neurology, and Rheumatology, Department of Internal Medicine, Kurume University School of Medicine, Kurume, Japan.

## Abstract

**Purpose:**

E7389-LF is a liposomal formulation of eribulin that contributes to tumor vascular remodeling. The phase II part of this phase Ib/II study assessed the efficacy/safety of E7389-LF in combination with nivolumab in several disease cohorts; herein, we report results from the small cell lung cancer (SCLC) cohort.

**Experimental Design:**

Patients with unresectable/measurable SCLC and disease progression with first-line platinum-based chemotherapy with/without an immune checkpoint inhibitor (ICI) were enrolled to receive E7389-LF 2.1 mg/m^2^ plus nivolumab 360 mg intravenously every 3 weeks. The primary objective of this part was to assess the objective response rate (ORR). Secondary objectives included assessments of safety and progression-free survival (PFS); exploratory assessments included overall survival (OS) and biomarkers.

**Results:**

Thirty-four patients were enrolled. By the data cut-off date (May 31, 2022), 29 (85.3%) had discontinued. Efficacy/biomarker analyses included 33 patients (1 had their diagnosis changed postenrollment); the ORR of E7389-LF plus nivolumab was 24.2% [95% confidence interval (CI): 11.1–42.3], the median PFS was 3.98 months (95% CI: 2.63–4.40), and, at a median follow-up of 10.6 months, the median OS was not reached (95% CI: not estimable). Notably, 27 of 33 patients (81.8%) had received an ICI as their prior first-line therapy. Treatment-related, treatment-emergent adverse events occurred in 97.1% (any grade) and 82.4% (grade ≥3) of enrolled patients; the most common event was neutropenia. Changes in vascular and immune-related plasma markers were observed.

**Conclusions:**

E7389-LF 2.1 mg/m^2^ in combination with nivolumab 360 mg every 3 weeks showed notable antitumor activity as second-line therapy for SCLC; no new safety signals were observed compared with either agent as monotherapy.

**Significance:**

This phase II part of a phase Ib/II study assessed liposomal eribulin (E7389-LF) plus nivolumab in 34 patients with pretreated SCLC; 8 of 33 evaluable patients (including 6/27 pretreated with ICIs) had objective responses. The combination was tolerable; increases in vasculature-related biomarkers tended to correlate with responses.

## Introduction

Lung cancer is among the most common cancers by incidence—in 2020, it represented 14% of new cancers in Japan and 10% of new cases in the United States, and resulted in approximately 20% of cancer deaths in both countries [International Agency for Research on Cancer, Global Cancer Observatory. (Cited 13 April 2023). Available from: https://gco.iarc.fr/today/data/factsheets/populations; RRID: SCR_012750]. In a registry study of 12,320 patients, small cell lung cancer (SCLC) represented approximately 19% of lung cancers in Japan and these patients had a 3-year survival rate of about 16% (1). Historically, chemotherapy has been the primary treatment for SCLC [National Comprehensive Cancer Network Clinical Practice Guidelines in Oncology (NCCN Guidelines). Non–small cell lung cancer. Version 2.2023. (Cited 13 April 2023). Available from: https://www.nccn.org/professionals/physician_gls/pdf/nscl.pdf; RRID: SCR_012959], while treatments for second-line therapy include topotecan (for platinum-sensitive SCLC; ref. 2) and amrubicin (in Japan; ref. 3). Typically, in patients with extensive-stage SCLC, an anti-programmed cell death ligand 1 (PD-L1) immune checkpoint inhibitor (ICI) is administered in combination with chemotherapies [National Comprehensive Cancer Network Clinical Practice Guidelines in Oncology (NCCN Guidelines). Non-Small Cell Lung Cancer. Version 2.2023. (Cited 13 April 2023). Available from: https://www.nccn.org/professionals/physician_gls/pdf/nscl.pdf; RRID: SCR_012959]. With the emergence of ICIs as part of primary therapy for SCLC, treatment strategies are needed for patients who fail to respond to ICIs.

Previously, nivolumab [an anti-programmed cell death 1 (PD-1) ICI] had been studied as monotherapy for patients with pretreated SCLC; however, it did not prove to be superior to the control (4). The combination of eribulin mesylate (a microtubule-dynamics inhibitor) with an anti-PD-1 antibody showed antitumor activity in a preclinical mouse model (5). E7389-LF is a liposomal formulation of eribulin mesylate (6). E7389-LF has previously shown efficacy and vascular remodeling effects in patients with advanced solid tumors in the dose-escalation part of the phase I Study 114 (7). Particularly, serum levels of IFNγ and levels of vasculature-related biomarkers, including collagen IV, tyrosine kinase immunoglobulin and EGF homology domains 2 [TIE2 (also known as TEK, TEK receptor tyrosine kinase)], intercellular adhesion molecule 1 (ICAM1), platelet endothelial cell adhesion molecule 1 (PECAM1), and VEGFR3, increased from cycle 1 day 1 (7). In the dose-expansion part of Study 114, E7389-LF monotherapy had a best overall response of stable disease in 6 of the 10 enrolled patients with SCLC, resulting in a disease control rate (DCR) of 60% (8). In a preclinical study, E7389-LF had improved *in vitro* immunomodulatory and antitumor activity versus eribulin mesylate, when both were used in combination with an anti-PD-1 antibody (9).

Study 120, an open-label phase Ib/II trial, was conducted to evaluate the potential of E7389-LF in combination with nivolumab in patients with various solid tumors. The phase Ib part evaluated the dosing and safety of E7389-LF + nivolumab in patients with solid tumors to determine a recommended phase II dose (10). The phase II part assessed efficacy and safety in cohorts of patients with gastric cancer, esophageal cancer, and SCLC. Herein, we report the efficacy and safety of E7389-LF plus nivolumab from the SCLC cohort of the phase II part of Study 120. The data cut-off date for the primary analysis was May 31, 2022—18 weeks after the last enrolled patient's first dose, as defined by the protocol.

## Materials and Methods

### Study Design and Patients

Patients with unresectable and measurable SCLC, who had been previously treated with combination therapy including a platinum agent with or without an ICI, were enrolled in the SCLC cohort of Study 120, an open-label, single-arm study (NCT04078295). Patients were treated with the recommended phase II dose of the phase Ib part: E7389-LF 2.1 mg/m^2^ plus nivolumab 360 mg administered intravenously every 3 weeks, in 21-day cycles. If treatment-related treatment-emergent adverse events (TEAE) occurred, the dose of E7389-LF could be reduced in consecutive steps to 1.7 mg/m^2^, 1.1 mg/m^2^, and then 0.8 mg/m^2^. Dose reductions of nivolumab were not permitted.

This study was conducted in accordance with standard operating procedures of the sponsor, which were based on the Principles of the World Medical Association Declaration of Helsinki, all applicable Japanese Good Clinical Practices and regulations, and the Pharmaceutical and Medical Device Act for studies conducted in Japan. Ethical approval and written informed consent were granted and approved by the applicable local Institutional Review Board. Signed informed consent forms were obtained from each patient prior to enrollment.

Enrolled patients were required to have at least one measurable lesion based on RECIST version 1.1 (RECIST v1.1). Lesions previously treated with radiotherapy or locoregional therapies must have shown evidence of progressive disease to be deemed a measurable lesion. Patients needed to have an Eastern Cooperative Oncology Group performance status (ECOG PS) of 0 or 1. Patients who had previous treatment with any anti-PD-1, anti-PD-L1, anti-PD-L2, anti-CD137, or anti-CTLA-4 antibody, or any other antibody or drug specifically targeting T-cell costimulation or checkpoint, or cancer vaccine therapy that resulted in a grade ≥3 immune-related adverse event, or who needed to discontinue treatment because of an immune-related adverse event of any grade, were excluded from the study. Additional inclusion and exclusion criteria are listed in the Supplementary Materials and Methods.

### Objectives and Assessments

The primary objective of the phase II part of Study 120 was to evaluate objective response rate (ORR) in patients who received E7389-LF plus nivolumab. Key secondary objectives included assessment of safety and progression-free survival (PFS). Exploratory objectives included assessment of DCR, overall survival (OS), and evaluation of potential biomarkers.

Tumor responses were assessed every 6 weeks by the investigators per RECIST v1.1. Safety assessments consisted of monitoring and recording all adverse events, including TEAEs; grading was conducted according to Common Terminology Criteria for Adverse Events–NCI version 5.0. Patient's survival was confirmed every 3 months (±2 weeks) from the time of treatment discontinuation and at the time of sponsor's request, until the time of treatment discontinuation for the last enrolled patient or 12 months after enrollment of the last case (whichever came later). Patients could continue to receive study drugs beyond the point of disease progression if they had investigator-assessed clinical benefit and were tolerating study drugs.

Patients were allowed to receive prophylactic pegylated GCSF (peg-GCSF) for the prevention of febrile neutropenia. For biomarker analyses, blood samples were collected as follows: for the once-every-3-weeks schedule, blood samples were collected predose on days (D) 1, 8, and 15 of cycles (C) 1 and 2, and day 1 of subsequent cycles (until cycle 9), and off-treatment. Seventy-eight biomarkers were investigated with AngiogenesisMAP, Multiplex, and Simoa systems. PD-L1 expression [by an approved IHC 28-8 pharmDx assay (Agilent Dako, Agilent Technologies)] and PD-L1 combined positive score (%, based on PD-L1 expression in viable tumor cells and immune cells) were assessed in tumor samples.

### Statistical Analyses

The primary objective of the phase II part was to assess ORR; the sample size was defined as a Bayesian posterior probability over 85% beyond threshold (15%) for ORR, corresponding to 7 or more responders among a planned population of 30 patients. The Safety Analysis Set included all patients who received at least one dose of E7389-LF or nivolumab (*N* = 34). The Efficacy and Biomarker Analysis Sets included 33 patients; 1 patient was excluded as they were later diagnosed with a different cancer than SCLC after enrollment.

ORR was calculated as proportion of patients with a complete response or partial response (PR); DCR was calculated as proportion of patients with a complete response, PR, or stable disease (for ≥5 weeks after first dose). Patients’ best overall responses of complete or PR required confirmation by a subsequent assessment of response at least 28 days later. PFS was defined as the time from the date of first dose to the date of first documented progressive disease or death due to any cause (whichever occurs first). For patients who did not have an event, PFS was censored. OS was defined as the time from the date of first study dose to the date of death from any cause. Patients who were lost to follow-up or who were alive at the date of data cutoff were censored at the date they were last known alive or the cut-off date, whichever came first.

Clopper–Pearson exact method was used to calculate 95% confidence intervals (CI) for ORR and DCR. For PFS and OS, medians were estimated by the Kaplan–Meier method and 95% CIs were calculated based on Greenwood formula. Estimates for median survival follow-up duration were calculated in the same way as the Kaplan–Meier estimates of OS but with the meaning of “censor” and “event” status indicators reversed. Patients’ relative dose intensities were calculated as the percentage of study drug received by patients over the planned dose (E7389-LF 2.1 mg/m^2^ plus nivolumab 360 mg administered intravenously every 3 weeks). Percentage changes in plasma biomarker levels from baseline were summarized using medians and analyzed using the Wilcoxon signed-rank test for each timepoint, and FDR adjustments (Benjamini–Hochberg) were also utilized. Wilcoxon rank-sum tests were also used to compare pharmacodynamic changes of vasculature or IFNγ-related biomarkers from baseline with best overall response [(complete or PR) vs. (stable or progressive disease)]. Statistical analyses were performed using SAS software (version 9.4 or later, SAS Institute).

### Data Availability Statement

Eisai Inc. commits to sharing data from clinical trials upon request from qualified scientific and medical researchers. Data requests are reviewed and authorized by an independent review panel on the basis of scientific merit, and data are anonymized with respect to applicable laws and regulations. Trial data availability is according to the criteria and process described here: https://www.clinicalstudydatarequest.com/SearchAllPostings.aspx.

## Results

### Patients

A total of 49 patients were screened for enrollment; 34 patients were enrolled and received treatment (Supplementary Fig. S1). Of the 34 patients who entered the treatment phase, 24 were men and 10 were women, and the median age was 66.0 years; Table 1 provides the baseline characteristics. The representativeness of patients in this cohort within a broader population of patients with SCLC is detailed in Supplementary Table S1. Of the 33 patients evaluable for efficacy analyses, 27 (81.8%) had received an ICI as prior first-line therapy and 13 (39.4%) received prophylactic peg-GCSF in cycle 1. A total of 17 patients had platinum-sensitive disease, defined as a progression-free interval of ≥90 days after completion of platinum therapy and 16 patients had platinum-resistant disease, defined as a progression-free interval of <90 days after completion of platinum therapy. Five patients had brain metastases at baseline; all had previously received radiotherapy. Additional characteristics of the evaluable population are provided in Supplementary Table S2.

**TABLE 1:** Patient demographics and baseline characteristics

Characteristic	Patients (*N* = 34)
Age, years, median (range)	66.0 (46–81)
Sex, *n* (%)	
Men	24 (70.6)
Women	10 (29.4)
Race, *n* (%)	
Japanese	34 (100)
Eastern Cooperative Oncology Group performance status, *n* (%)	
0	15 (44.1)
1	19 (55.9)
Median weight, kg (range)	64.40 (42.4–86.2)
Median body surface area, m^2^ (range)	1.722 (1.30–2.03)
Brain metastases at baseline, *n* (%)	5 (14.7)
Number of previous anticancer medication regimens (excluding adjuvant/neo-adjuvant), patient *n* (%)	
1	34 (100.0)^a^
Previous therapies for advanced/metastatic disease, *n* (%)	
Etoposide	32 (94.1)
Carboplatin	22 (64.7)
Atezolizumab	16 (47.1)
Cisplatin	15 (44.1)
Durvalumab	8 (23.5)
Investigational drug^b^	6 (17.6)
Pembrolizumab	2 (5.9)
Vibostolimab	2 (5.9)
Immune checkpoint inhibitor	1 (2.9)
Irinotecan	1 (2.9)
Ramucirumab	1 (2.9)
Topoisomerase 1 inhibitor	1 (2.9)

^a^Two patients (5.6%) also received one line of adjuvant/neoadjuvant therapy, and thus had two regimens of prior anticancer therapy.

^b^Only includes drugs received as part of a clinical study which could not be identified.

By the data cut-off date (May 31, 2022), 5 of the 34 patients enrolled (14.7%) were still undergoing treatment. Discontinuations occurred in 29 patients (85.3%); primarily due to disease progression in 26 patients (76.5%) and to an adverse event in 3 patients (8.8%).

### Efficacy

**FIGURE 1:**
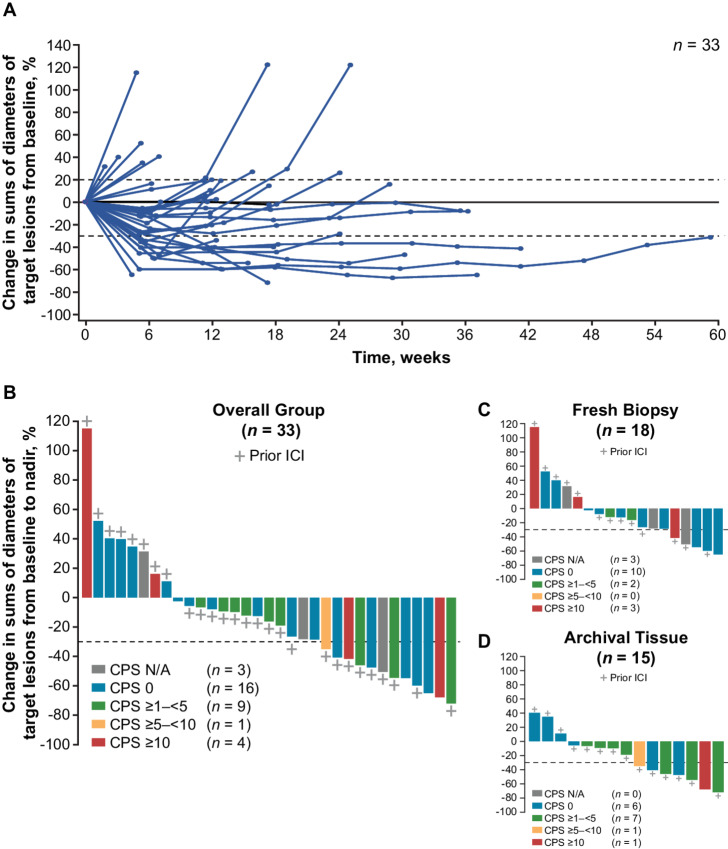
Percentage changes in sums of diameters of target lesions from baseline over time (**A**)^a^, and by PD-L1 CPS at nadir in all patients (**B**), patients with a biopsy (**C**), and patients with an archival tumor sample (**D**) ^a^Patients could continue to receive study drugs beyond disease progression if they had investigator-assessed clinical benefit and were tolerating study drugs. A includes data from beyond disease progression. CPS, combined positive score; ICI, immune checkpoint inhibitor; N/A, not available; PD-L1, programmed cell death ligand 1.

Of the 33 patients evaluable for efficacy analyses, 8 patients had a PR and 17 had stable disease; thus E7389-LF + nivolumab had an ORR of 24.2% (95% CI: 11.1–42.3) and a DCR of 75.8% (95% CI: 57.7–88.9; Supplementary Table S2). The Bayesian posterior probability for ORR was 94.1%, and was thus beyond the planned threshold of 85%. Of the 27 patients whose first-line therapy included an ICI, the ORR was 22.2% (95% CI: 8.6–42.3) and the DCR was 74.1% (95% CI: 53.7–88.9). The ORR of platinum-sensitive patients was 41.2% (*n* = 7/17), while the ORR of platinum-resistant patients was 6.3% (*n* = 1/16). The ORR of the 13 patients who received prophylactic peg-GCSF in cycle 1 was 38.5% compared with 15.0% of the 20 patients who did not. The ORR and DCR of the 5 patients with baseline brain metastases were 20.0% and 60.0% compared with 25.0% and 78.6% in the 28 patients who did not have brain metastases (Supplementary Table S2); notably, none of these 5 patients had progressive disease in their brain lesions; however, an additional 3 patients developed brain metastases during the study and had progressive disease. ORR and DCR by additional characteristics including disease stage, ECOG PS, and baseline lactic dehydrogenase are reported in Supplementary Table S2. Changes in the size of target lesions over time are shown in Fig. 1A; tumor reductions at nadir were observed irrespective of PD-L1 expression, including in patients with a PD-L1 combined positive score of 0 (Fig. 1B).

**FIGURE 2:**
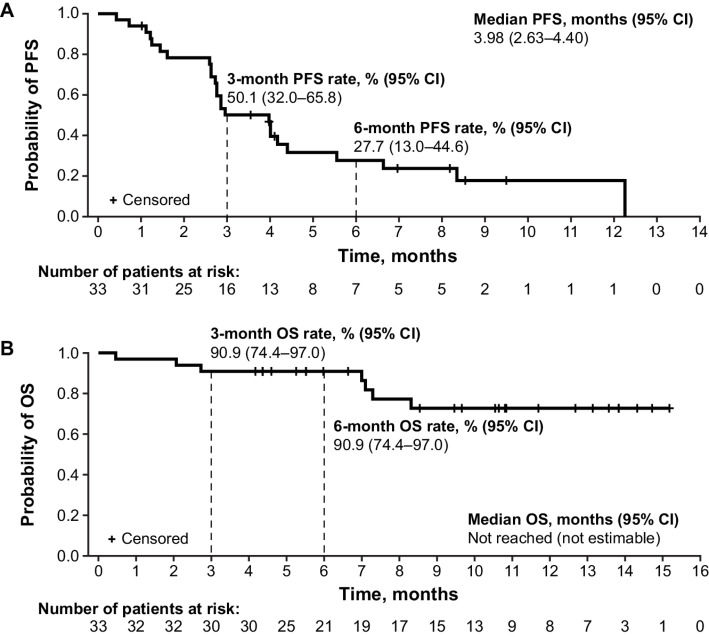
Kaplan–Meier Curves of PFS (**A**) and OS (**B**). CI, confidence interval; OS, overall survival; PFS, progression-free survival.

The median PFS was 3.98 months (95% CI: 2.63–4.40); the 3-month PFS rate was 50.1% (95% CI: 32.0–65.8) and the 6-month PFS rate was 27.7% (95% CI: 13.0–44.6; Fig. 2A). In the 27 patients who received an ICI as first-line therapy, the median PFS was 2.86 months (95% CI: 2.60–4.40), the 3-month PFS rate was 40.7% (95% CI: 22.5–58.2), and the 6-month PFS rate was 21.4% (95% CI: 7.5–39.9). The median PFS was 4.40 months (95% CI: 2.96–12.25) in platinum-sensitive patients and 2.68 months (95% CI: 1.22–2.86) in platinum-resistant patients; the 3-month PFS rates were 75.0% (95% CI: 46.3–89.8) and 25.0% (95% CI: 7.8–47.2), and the 6-month PFS rates were 43.3% (95% CI: 17.2–67.2) and were 12.5% (95% CI: 2.1–32.8), respectively. In the 13 patients who received prophylactic peg-GCSF in cycle 1, the median PFS was 8.34 months [95% CI: 1.61–not estimable (NE)], the 3-month PFS rate was 69.2% (95% CI: 37.3–87.2), and the 6-month PFS rate was 50.5% (95% CI: 20.6–74.4). In the 20 patients who did not receive prophylactic peg-GCSF, the median PFS was 2.76 months (95% CI: 2.60–4.17), the 3-month PFS rate was 37.1% (95% CI: 16.7–57.7), and the 6-month PFS rate was 15.9% (95% CI: 3.9–35.1). In the 5 patients who had brain metastases at baseline, the median PFS was 2.63 months (95% CI: 0.72–NE) and the 3-month PFS rate was 20.0% (95% CI: 0.8–58.2). In the 28 patients who did not have brain metastases at baseline, the median PFS was 4.01 months (95% CI: 2.73–5.55), the 3-month PFS rate was 55.6% (95% CI: 35.3–71.9), and the 6-month PFS rate was 30.6% (14.1–48.8).

The median survival follow-up duration for OS was 10.6 months; the median OS was not reached (NR; 95% CI: NE) and the 3- and 6-month OS rates were both 90.9% (95% CI: 74.4–97.0; Fig. 2B). In the 27 patients who received a prior ICI, the median OS was NR (95% CI: 7.29–NE), and the 3- and 6-month OS rates were both 88.9% (95% CI: 69.4–96.3). In platinum-sensitive patients, the median OS was NR (95% CI: NE); the 3- and 6-month OS rates were both 100% (95% CI: 100–100). The median OS of platinum-resistant patients was 8.31 months (95% CI: 7.00–NE); the 3- and 6-month OS rates were both 81.3% (95% CI: 52.5–93.5). Similarly, in the 13 patients who received peg-GCSF, the median OS was NR (95% CI: 7.00 months–NE), and the 3- and 6-month OS rates were 100% (95% CI: 100–100). In the 20 patients who did not receive peg-GCSF, the median OS was NR (95% CI: 8.31 months–NE), and the 3- and 6-month OS rates were both 85.0% (95% CI: 60.4–94.9). In the 5 patients who had brain metastases at baseline, the median OS was 7.10 months (95% CI: 2.07–NE), the 3-month OS rate was 60.0% (95% CI: 12.6–88.2), and the 6-month OS rate was 60.0% (95% CI: 12.6–88.2). In the 28 patients who did not have brain metastases at baseline, the median OS was NR (95% CI: NR), the 3- and 6-month OS rates were 96.4% (95% CI: 77.2–99.5).

As the median OS was NR by the data cut-off date of the primary analysis, an extended analysis was conducted 12 months after the last enrolled patient's first dose (i.e., January 25, 2023). At the median survival follow-up duration of 20.53 months, the median OS was 12.86 months (95% CI: 10.94–18.92; Supplementary Fig. S2).

### Dose Intensity

**FIGURE 3:**
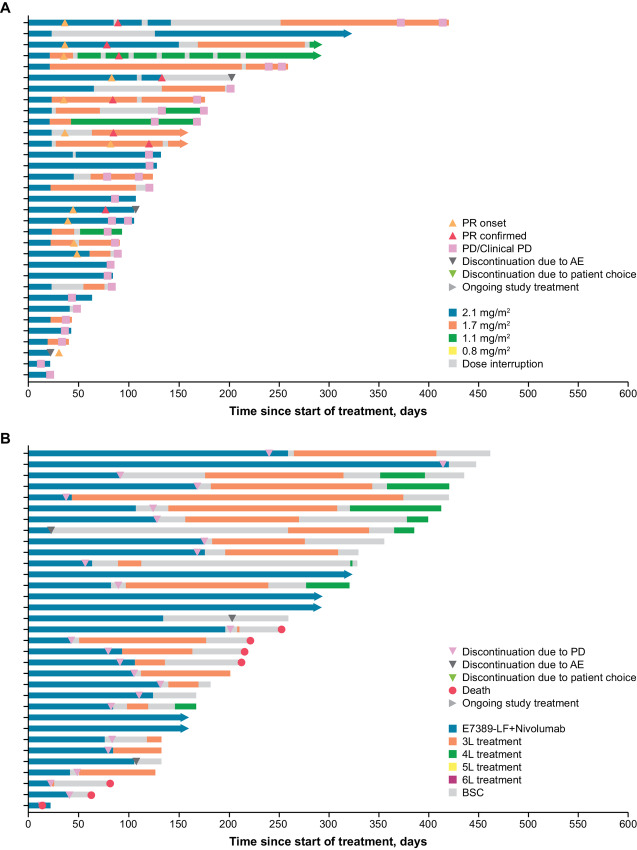
Swimmer plots of patients by course of treatment and dose reduction of E7389-LF (**A**) and lines of subsequent therapies received during survival follow-up (**B**). Patients could continue to receive study drugs beyond disease progression if they had investigator-assessed clinical benefit and were tolerating study drugs. This figure includes data from beyond disease progression. #L, line of treatment number; AE, adverse event; BSC, best supportive care; PD, progressive disease; PR, partial response.

Doses and dose reductions of study treatment received over time for the 33 patients evaluable for efficacy analyses are shown in Fig. 3A. PRs were generally sustained through dose reductions of E7389-LF (i.e., 1.7 and 1.1 mg/m^2^). Of the 33 evaluable patients, 28 discontinued study treatment, of whom 22 received subsequent anticancer therapy during survival follow-up (Supplementary Table S3; Fig. 3B). In the 33 patients evaluable for efficacy analyses, the relative dose intensity of E7389-LF was 69.31%; the relative dose intensity of nivolumab was 72.57%. Relative dose intensities of E7389-LF were 73.05% in patients who received peg-GCSF and 65.98% in those who did not receive peg-GCSF. Relative dose intensities of nivolumab were 71.70% in patients who received peg-GCSF and 73.33% in patients who did not receive peg-GCSF.

### Safety

Treatment-related TEAEs of any grade and of grade ≥3 occurred in 97.1% and 82.4% of the 34 enrolled patients, respectively (Table 2). The most common treatment-related TEAEs were neutropenia (58.8%), leukopenia (55.9%), and decreased appetite (55.9%). The most common grade ≥3 treatment-related TEAEs were neutropenia (52.9%), leukopenia (44.1%), and febrile neutropenia (26.5%). Of the 21 patients who did not receive prophylactic peg-GCSF, 8 (38.1%) had febrile neutropenia; 1 of the 13 patients (7.7%) who received peg-GCSF had febrile neutropenia. Generally, the neutrophil count nadir occurred around C1D8 and C1D15 in patients who did not receive prophylactic peg-GCSF.

**TABLE 2:** TEAEs that occurred in >10% of patients

	Patients (*N* = 34)			
	Treatment-related TEAES		TEAEs of any cause	
MedDRA preferred term, *n* (%)	Any grade	Grade ≥3	Any grade	Grade ≥3
Patients with any TEAEs	33 (97.1)	29 (82.4)	34 (100)	29 (85.3)
Neutropenia	20 (58.8)	18 (52.9)	20 (58.8)	18 (52.9)
Leukopenia	19 (55.9)	15 (44.1)	19 (55.9)	15 (44.1)
Decreased appetite	19 (55.9)	3 (8.8)	20 (58.8)	3 (8.8)
Malaise	15 (44.1)	1 (2.9)	15 (44.1)	1 (2.9)
Aspartate aminotransferase increased	11 (32.4)	1 (2.9)	11 (32.4)	1 (2.9)
Nausea	10 (29.4)	0	11 (32.4)	0
Alanine aminotransferase increased	9 (26.5)	0	9 (26.5)	0
Febrile neutropenia	9 (26.5)	9 (26.5)	9 (26.5)	9 (26.5)
Thrombocytopenia	9 (26.5)	5 (14.7)	9 (26.5)	5 (14.7)
Pneumonitis	7 (20.6)	2 (5.9)	7 (20.6)	0
Infusion-related reaction	6 (17.6)	0	6 (17.6)	0
Pyrexia	6 (17.6)	0	11 (32.4)	0
Anemia	5 (14.7)	0	7 (20.6)	1 (2.9)
Pruritus	5 (14.7)	1 (2.9)	6 (17.6)	1 (2.9)
Alopecia	4 (11.8)	0	4 (11.8)	0
Dysgeusia	4 (11.8)	0	4 (11.8)	0
Gamma-glutamyltransferase increased	4 (11.8)	0	5 (14.7)	0
Lymphopenia	4 (11.8)	2 (5.9)	4 (11.8)	2 (5.9)
Peripheral sensory neuropathy	4 (11.8)	0	5 (14.7)	0
Rash	4 (11.8)	0	4 (11.8)	0
Constipation	3 (8.8)	0	8 (23.5)	0
Pneumonia	3 (8.8)	2 (5.9)	5 (14.7)	2 (5.9)
Proteinuria	3 (8.8)	0	4 (11.8)	0
Rash maculopapular	3 (8.8)	0	4 (11.8)	0
Stomatitis	3 (8.8)	0	5 (14.7)	0
Urticaria	3 (8.8)	1 (2.9)	4 (11.8)	1 (2.9)
Abdominal pain upper	2 (5.9)	0	4 (11.8)	0
Cough	2 (5.9)	0	8 (23.5)	1 (2.9)
Diarrhea	2 (5.9)	0	4 (11.8)	0
Hypokalemia	2 (5.9)	0	5 (14.7)	0
Insomnia	0	0	5 (14.7)	0

Abbreviation: TEAE, treatment-emergent adverse event.

Overall, all patients had TEAEs of any grade and 85.3% had grade ≥3 TEAEs; the most common of which were similar to the most common types of treatment-related TEAEs (Table 2). One patient had grade 5 septic shock unrelated to either study medication. TEAEs led to dose reduction of E7389-LF in 19 patients (55.9%), most commonly febrile neutropenia that occurred in 8 patients (23.5%). Withdrawal of either E7389-LF or nivolumab occurred in 5 patients (14.7%): causes were acute kidney injury, cough, myocarditis, pneumonitis, and radiation pneumonitis (*n* = 1 each).

### Biomarker Analyses

**FIGURE 4:**
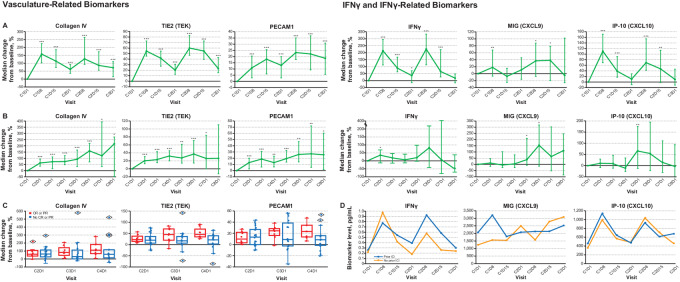
Changes from baseline in select vasculature and IFN-related biomarkers weekly to C3D1 (**A**), per cycle to C8D1 (**B**), by tumor response (for vasculature-related biomarkers; **C**), and by presence of prior ICI treatment (for IFN-related biomarkers; **D**). Lines represent medians; error bars represent 95% CI. For box-and-whisker plots in C, the horizontal line represents the median, the box represents the interquartile range, the whiskers represent the largest or smallest values within 1.5 times the interquartile range (either above the 75th percentile or below the 25th percentile), and diamonds represent outliers. *P* values are: *, *P* < 0.05; **, *P* < 0.01; ***, *P* < 0.001; patient numbers are as follows: **A**: *n* = 33 (C1D1, C1D8), *n* = 32 (C1D15), *n* = 30 (C2D1), *n* = 29 (C2D8), *n* = 28 (C2D15), *n* = 25 (C3D1). **B**: *n* = 33 (C1D1). *n* = 30 (C2D1), *n* = 25 (C3D1), *n* = 24 (C4D1), *n* = 19 (C5D1), *n* = 12 (C6D1), *n* = 8 (C7D1), *n* = 7 (C8D1). **C**: for CR/PR, *n* = 8 (C2D1, C3D1, C4D1); for non-CR/PR, *n* = 22 (C2D1), *n* = 17 (C3D1), *n* = 16 (C4D1). **D**: for prior ICI, *n* = 27 (C1D1, C1D8), *n* = 26 (C1D15), *n* = 25 (C2D1, C2D8, C2D15), *n* = 20 (C3D1); for no prior ICI, *n* = 6 (C1D1, C1D8, C1D15), *n* = 5 (C2D1), *n* = 4 (C2D8), *n* = 3 (C2D15), *n* = 5 (C3D1). C#D#, cycle #, day; CR, complete response; ICI, immune checkpoint inhibitor; IFNγ, interferon gamma; IP-10, IFN gamma-induced protein 10; MIG, monokine induced by gamma IFN; PECAM1, platelet endothelial cell adhesion molecule 1; PR, partial response; TEK, TEK receptor tyrosine kinase; TIE2, tyrosine kinase immunoglobulin and EGF homology domains 2.

Of the 78 biomarkers tested for plasma biomarker analysis, 49 were considered evaluable because ≥70% of samples had levels above the lower limit of quantification for biomarker analyses. Clear increases from baseline in levels of IFNγ, IFNγ-related markers monokine induced by gamma IFN [MIG; also known as CXCL9 (c-x-c motif chemokine ligand 9)], IFNγ-induced protein 10 (IP-10; also called CXCL10), IFN-inducible T-cell alpha chemoattractant (ITAC, also called CXCL11) and six of seven vasculature-related markers (i.e., collagen IV, TIE2, ICAM1, PECAM1, VEGFR3, and endoglin) were observed (Fig. 4; Supplementary Table S4; Supplementary Fig. S3). Peak changes occurred at days 8–15 of each cycle (Fig. 4A; Supplementary Fig. S3A). Changes in IFNγ, MIG, ITAC, collagen IV, TIE2, ICAM1, PECAM1, and VEGFR3 were also seen at later treatment cycles (Fig. 4B; Supplementary Fig. S3B). Changes in biomarkers, including collagen IV, TIE2, and PECAM 1 were more apparent in those patients who had a response (Fig. 4C; Supplementary Table S5). Changes in IFNγ and related signaling biomarkers MIG and IP-10 were observed irrespective of receipt of a prior ICI (Fig. 4D); baseline levels of these biomarkers by receipt of prior ICI are shown in Supplementary Fig. S4.

## Discussion

E7389-LF in combination with nivolumab was well tolerated and demonstrated anticancer activity as second-line therapy for patients with SCLC. The overall ORR was 24.2%, which exceeded the planned criteria. PRs were generally sustained despite any dose reductions, suggesting that the starting dose and dose-reduction rules were appropriate. Acknowledging the limitations of cross-study comparisons, the small study population size, and the fact that patients in previous trials generally did not receive ICIs as prior therapy, this overall ORR appeared promising compared with the ORRs observed in the CheckMate 331 study (nivolumab: 13.7%; topotecan or amrubicin chemotherapy: 16.5%) for patients with pretreated SCLC (4). The ORR was comparable to a phase III trial of amrubicin (31.1%) versus topotecan (16.9%), although it should be noted that efficacy was evaluated per RECIST v1.0 instead of v1.1 (11). Finally, the ORR was also comparable to a phase I study of tarlatamab (23.4%), where 50% of the enrolled population had received a PD-1 inhibitor in addition to chemotherapy, (12) although tarlatamab is currently undergoing testing in a phase II trial (NCT05060016).

E7389-LF in combination with nivolumab had a median PFS of 3.98 months and 6-month PFS rate of 27.7%; this was promising given the outcomes of the CheckMate 331 study of nivolumab, which reported a median PFS of 1.4 months and a 6-month PFS rate of 19.7% (4). In addition, the 6-month OS rate of the combination (90.9%) appeared to be much higher than that in the trial of nivolumab (54.5%) or topotecan/amrubicin (59.9%; ref. 4). OS was NR at a median survival follow-up duration of 10.6 months because most patients (22 of the 28 that discontinued) had received subsequent therapy; this suggests that E7389-LF + nivolumab did not negatively impact the treatment period of these subsequent therapies. As median OS data were premature at the May 31, 2022 data cutoff, we conducted additional follow-up and determined that the median OS was 12.86 months as of January 25, 2023.

In patients pretreated with an ICI, the ORR was 22.2%, and the median OS was NR as of May 31, 2022. These results are promising, as second-line treatment options are needed for patients with SCLC who received an ICI in the first-line. The FDA has approved lurbinectedin for adult patients whose metastatic SCLC has progressed on or after platinum-based chemotherapy under accelerated approval based on a single-arm phase II study, which reported an ORR of 35.2% (13, 14); however, a minority of patients (8%) received an ICI before enrollment. Thus, options for patients who were previously treated with an ICI remain limited. We find the results of this current analysis encouraging in supporting E7389-LF in combination with nivolumab as a potential option to address this unmet medical need.

In patients who received peg-GCSF, the ORR, PFS rates, and OS rates were notably higher than in patients who did not receive peg-GCSF. Patients who received peg-GCSF also had a higher relative dose intensity of E7389-LF than patients without peg-GCSF, likely because peg-GCSF prevented cases of febrile neutropenia that would otherwise have affected dosing. In addition, patients who had a progression-free interval of ≥90 days because completion of their first-line platinum therapy had a particularly high ORR (41.2%) and DCR (88.2%).

No new safety signals were observed compared with the known profiles of each monotherapy (15, 16). The incidences of treatment-related leukopenia and neutropenia were similar to those in the phase Ib part of this study (10). Of note, more patients in this cohort (13/34 patients, 38.2%) received peg-GCSF than in the phase Ib part (4/25 patients, 16.0%; ref. 10).

Plasma levels of vasculature- and IFNγ-related biomarkers increased during treatment, suggesting vascular remodeling and immune response activation. These results are consistent with the phase Ib part of this study (10). Longer treatment increased the nadir level of collagen IV, TIE2, and PECAM1, particularly in those who had a response. Thus, these vascular markers may be helpful surrogates for biomarkers for clinical response by the combination. In addition, changes in biomarkers IFNγ and related markers MIG and IP-10 were seen regardless of receipt of a prior ICI, suggesting that immune response activation is not dictated by prior therapy.

However, our findings are limited by the single-arm, single-country (Japan) nature of this study, as well as the sample size (*n* = 34). The high incidence of prior ICI therapy also complicates comparisons to other studies; antitumor activity may have been affected by pre-existing ICI resistance that would not have been present in trials of ICI-naïve populations. Despite these shortcomings, the combination of E7389-LF and nivolumab may be a suitable option for patients with unresectable, pretreated SCLC, and should be evaluated further in larger, standardized populations.

## Supplementary Material

Supplemental Figure 1 Supplementary Figure 1. Patient Disposition

Supplemental Figure 2 Supplementary Figure 2. Kaplan–Meier Curve of OS 12 Months of Follow-up from the Last Patient’s First Dose (Data Cutoff Date: January 25, 2023)

Supplemental Figure 3 Supplementary Figure 3. Changes from Baseline in Additional Vasculature- and IFNγ-related Biomarkers Weekly to C3D1 (A), and Per Cycle to C8D1 (B)

Supplemental Figure 4 Supplementary Figure 4. IFN-related Biomarker Levels at Baseline by Receipt of Prior ICI

Supplemental Methods Supplemental Methods

Supplemental Table 1 Supplementary Table 1. Representativeness of Study Participants

Supplemental Table 2 Supplementary Table 2. Tumor Responses in Evaluable Patients, and by Baseline/Pretreatment Characteristics

Supplemental Table 3 Supplementary Table 3. Anticancer Therapies Received During Survival Follow-up

Supplemental Table 4 Supplementary Table 4. Median Change in Biomarkers from Baseline

Supplemental Table 5 Supplementary Table 5. Median Change in Biomarkers from Baseline Over Time by Tumor Response
