# Supplementary material for: Phase II Study of the Liposomal Formulation of Eribulin (E7389-LF) in Combination with Nivolumab: Results from the Small Cell Lung Cancer Cohort
Source: Cancer Res Commun. 2024 Jan 29;4(1):226–35. doi: 10.1158/2767-9764.CRC-23-0313 (PMC10823908; doi:10.1158/2767-9764.CRC-23-0313)
Supplement: Supplemental Figure 1 — Supplementary Figure 1. Patient Disposition [file crc-23-0313-s01.pdf]

Supplementary Figure 1. Patient Disposition

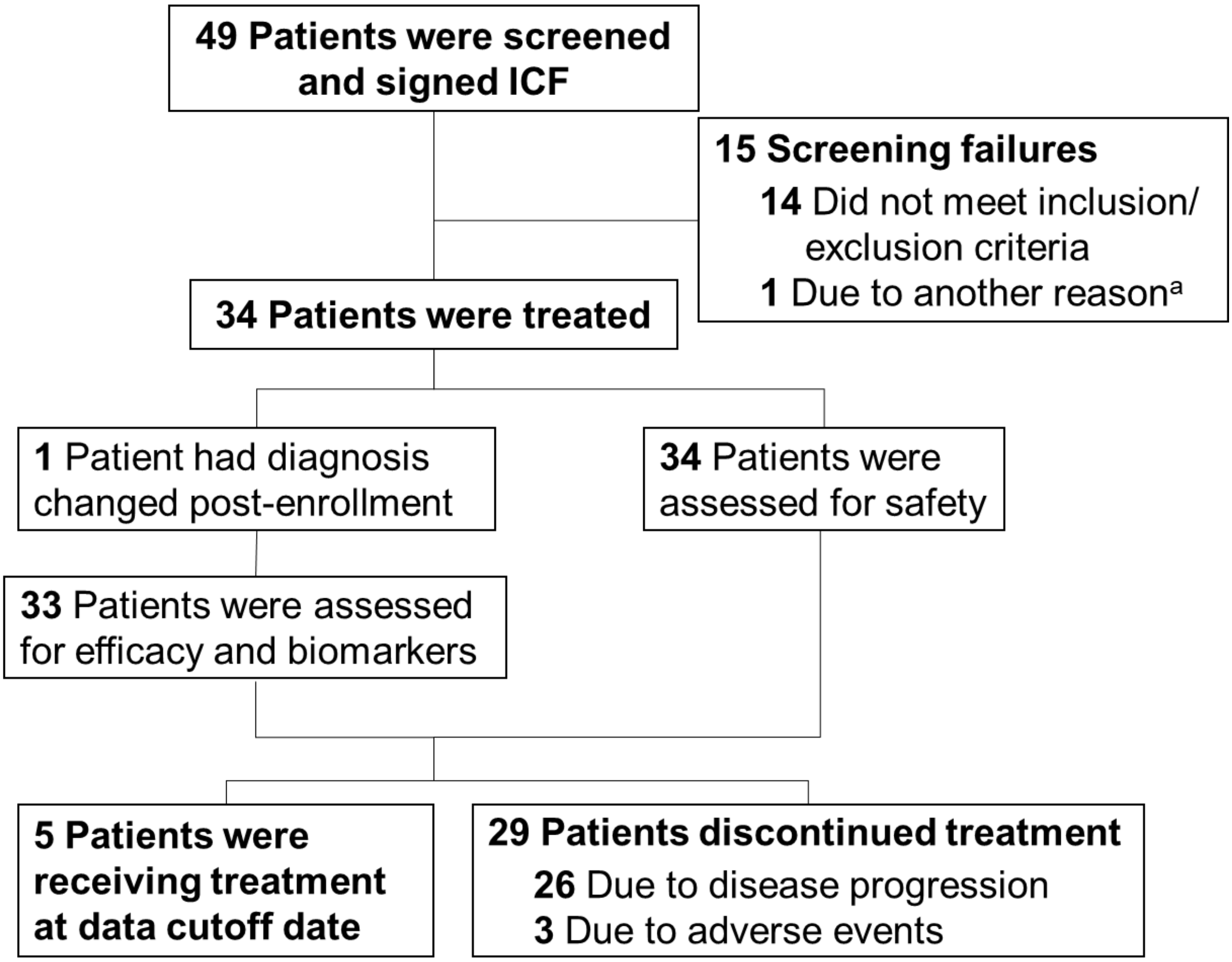

[a]Patient required urgent treatment and could not wait for screening period to finish.

ICF, informed consent form.
