# Supplementary material for: Phase II Study of the Liposomal Formulation of Eribulin (E7389-LF) in Combination with Nivolumab: Results from the Small Cell Lung Cancer Cohort
Source: Cancer Res Commun. 2024 Jan 29;4(1):226–35. doi: 10.1158/2767-9764.CRC-23-0313 (PMC10823908; doi:10.1158/2767-9764.CRC-23-0313)
Supplement: Supplemental Figure 2 — Supplementary Figure 2. Kaplan–Meier Curve of OS 12 Months of Follow-up from the Last Patient’s First Dose (Data Cutoff Date: January 25, 2023) [file crc-23-0313-s02.pdf]

Supplementary Figure 2. Kaplan–Meier Curve of OS 12 Months of Follow-up from the Last Patient's First Dose (Data Cutoff Date: January 25, 2023)

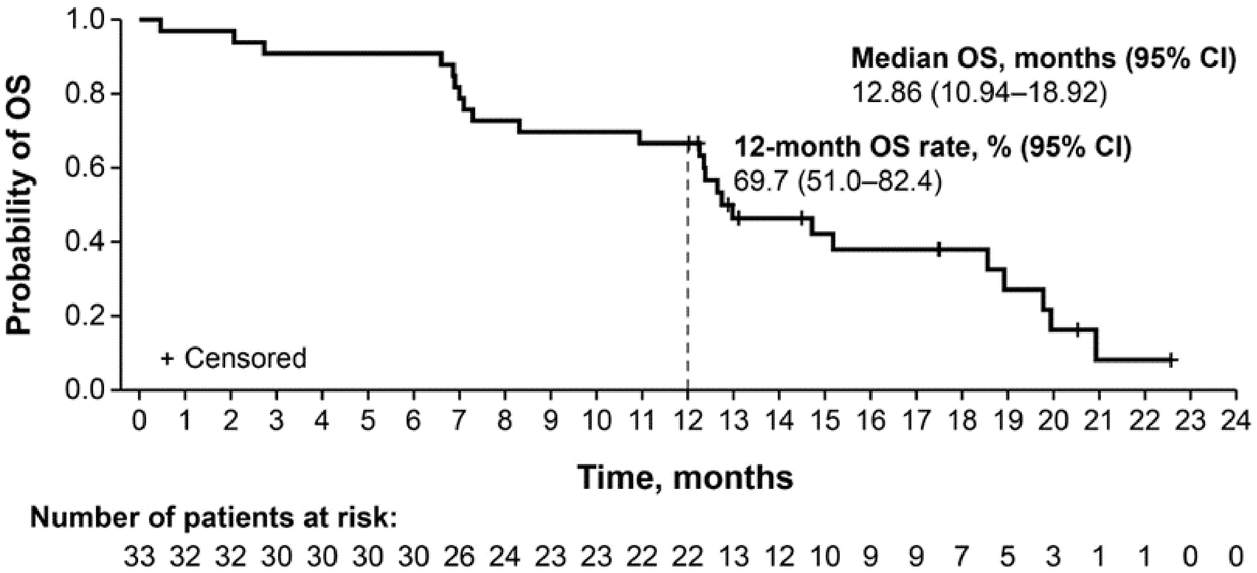

CI, confidence interval; OS, overall survival.
