# Supplementary material for: Phase II Study of the Liposomal Formulation of Eribulin (E7389-LF) in Combination with Nivolumab: Results from the Small Cell Lung Cancer Cohort
Source: Cancer Res Commun. 2024 Jan 29;4(1):226–35. doi: 10.1158/2767-9764.CRC-23-0313 (PMC10823908; doi:10.1158/2767-9764.CRC-23-0313)
Supplement: Supplemental Figure 3 — Supplementary Figure 3. Changes from Baseline in Additional Vasculature- and IFNγ-related Biomarkers Weekly to C3D1 (A), and Per Cycle to C8D1 (B) [file crc-23-0313-s03.pdf]

Supplementary Figure 3. Changes from Baseline in Additional Vasculature- and IFNγ-related Biomarkers Weekly to C3D1 (A), and Per Cycle to C8D1 (B)

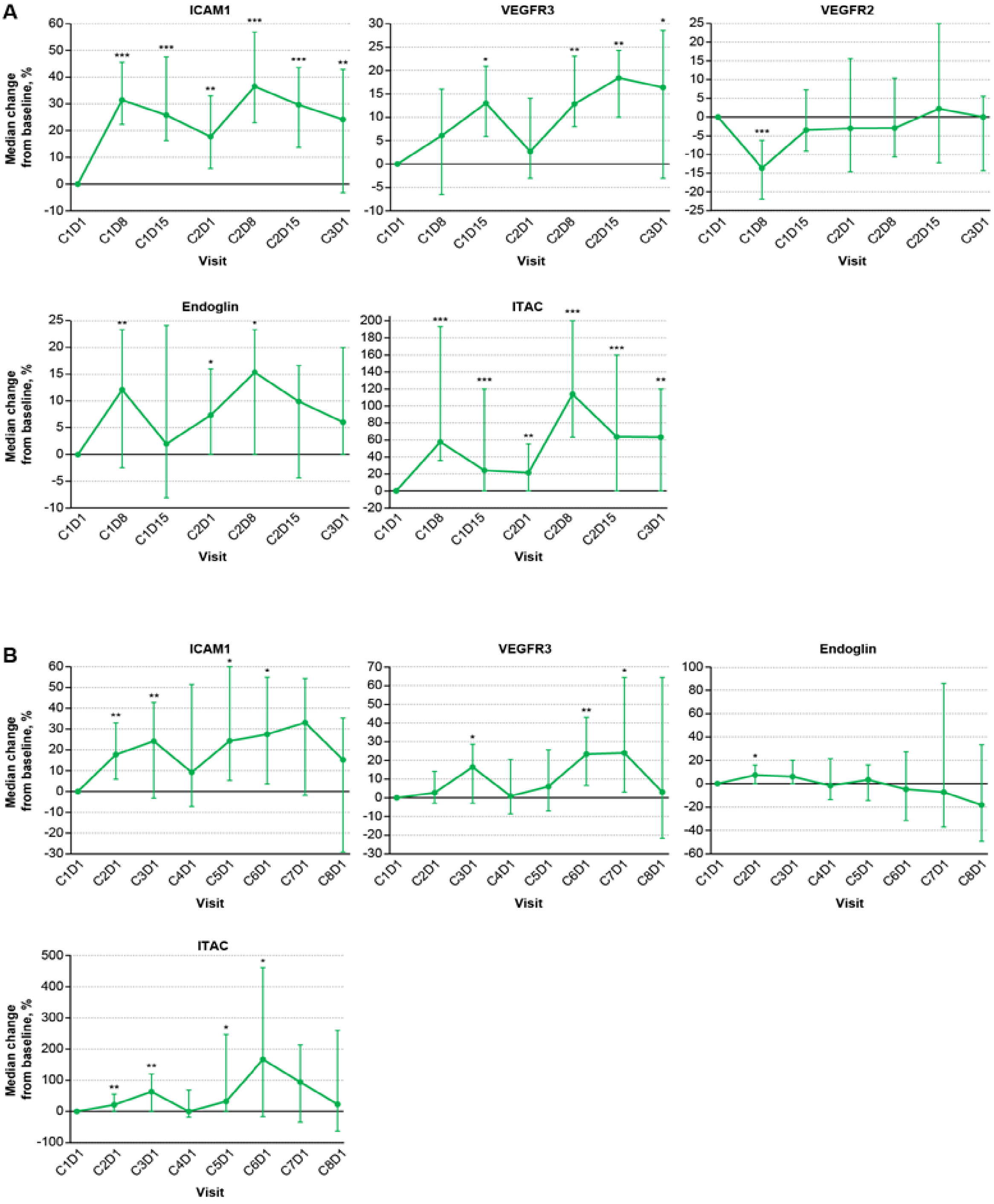

Lines represent medians, error bars represent 95% confidence interval. P-values: *P < 0.05, **P < 0.01, ***P < 0.001.
Patient numbers are as follows:
Part A: n=33 (C1D1, C1D8), n=32 (C1D15), n=30 (C2D1), n=29 (C2D8), n=28 (C2D15), n=25 (C3D1).
Part B: n=33 (C1D1). n=30 (C2D1), n=25 (C3D1), n=24 (C4D1), n=19 (C5D1), n=12 (C6D1), n=8 (C7D1), n=7 (C8D1).

C#D#, cycle #, day #; CXCL, c-x-c motif chemokine ligand; ICAM1, intercellular adhesion molecule 1; ITAC, interferon-inducible T alpha chemoattractant; VEGFR3, vascular endothelial growth factor receptor 3.
