# Supplementary material for: Phase II Study of the Liposomal Formulation of Eribulin (E7389-LF) in Combination with Nivolumab: Results from the Small Cell Lung Cancer Cohort
Source: Cancer Res Commun. 2024 Jan 29;4(1):226–35. doi: 10.1158/2767-9764.CRC-23-0313 (PMC10823908; doi:10.1158/2767-9764.CRC-23-0313)
Supplement: Supplemental Figure 4 — Supplementary Figure 4. IFN-related Biomarker Levels at Baseline by Receipt of Prior ICI [file crc-23-0313-s04.pdf]

Supplementary Figure 4. IFN-related Biomarker Levels at Baseline by Receipt of Prior ICI

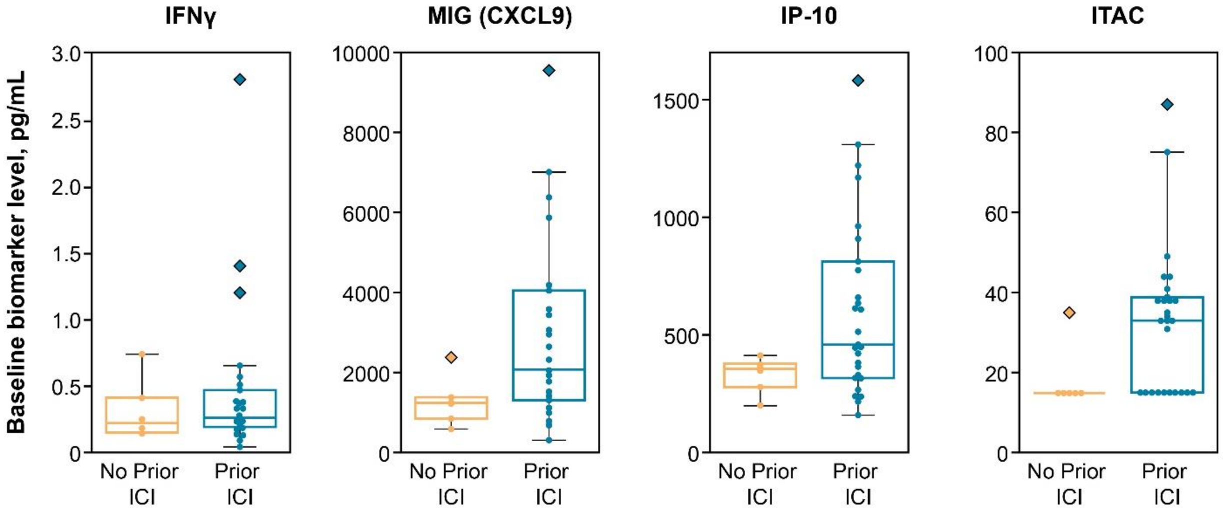

Horizontal lines represent median, boxes represent interquartile range, whiskers represent the largest/smallest values within 1.5 times the interquartile range (either above the 75th percentile or below the 25th percentile) and diamonds represent outliers. Patient counts are n=6 for no prior ICI treatment, and n=27 for prior ICI treatment.

CXCL, c-x-c motif chemokine ligand; ICI, immune checkpoint inhibitor; IFN, interferon; IP-10, interferon gamma-induced protein 10; ITAC, interferon-inducible T alpha chemoattractant; MIG, monokine induced by gamma interferon.
