# Supplementary material for: Phase II Study of the Liposomal Formulation of Eribulin (E7389-LF) in Combination with Nivolumab: Results from the Small Cell Lung Cancer Cohort
Source: Cancer Res Commun. 2024 Jan 29;4(1):226–35. doi: 10.1158/2767-9764.CRC-23-0313 (PMC10823908; doi:10.1158/2767-9764.CRC-23-0313)
Supplement: Supplemental Table 1 — Supplementary Table 1. Representativeness of Study Participants [file crc-23-0313-s06.pdf]

Supplementary Table 1. Representativeness of Study Participants

**Cancer Type**	Small cell lung cancer
**Considerations related to:**	
Sex	Most patients in our trial were male (70.6%) this generally corresponds to global data, where males have an age-standardized rate of lung cancer of 47.0, compared with 19.5 for women.[a]
Age	The median age of patients in our trial was 66.0 years, which aligns with real-world populations.[b]
Ethnicity/geography	All patients in our trial were Japanese. In Japan, lung cancer is the highest-ranked cancer in terms of incidence and mortality.[a]
**Overall representativeness of this study**	In general, several characteristics of patients in our study are consistent with adults (particularly Japanese adults) diagnosed with small cell lung cancer. Our findings should be validated in a larger sample size enrolling patients from different countries.

[a]Japan – Global Cancer Observatory. GLOBOCAN 2020. Accessed January 13, 2023. https://gco.iarc.fr/today/data/factsheets/populations/392-japan-fact-sheets.pdf.

[b]Lee MH, Qureshi MM, Suzuki, et al. Small cell lung cancer in young patients: trends in sociodemographic factors, diagnosis, treatment, and survival. *J Thorac Dis*. 2022;14(8):2880–2893.
