# Supplementary material for: Phase II Study of the Liposomal Formulation of Eribulin (E7389-LF) in Combination with Nivolumab: Results from the Small Cell Lung Cancer Cohort
Source: Cancer Res Commun. 2024 Jan 29;4(1):226–35. doi: 10.1158/2767-9764.CRC-23-0313 (PMC10823908; doi:10.1158/2767-9764.CRC-23-0313)
Supplement: Supplemental Table 2 — Supplementary Table 2. Tumor Responses in Evaluable Patients, and by Baseline/Pretreatment Characteristics [file crc-23-0313-s07.pdf]

Supplementary Table 2. Tumor Responses in Evaluable Patients, and by Baseline/Pretreatment Characteristics

Characteristic	Evaluable patients, n (n = 33)	Objective Response Rate		Disease Control Rate	
		n (%)	95% CI	Rate, n (%)	95% CI
**All patients**	33	8 (24.2)	11.1–42.3	25 (75.8)	57.7–88.9
**Patients with prior ICI treatment**					
Yes	27	6 (22.2)	8.6–42.3	20 (74.1)	53.7–88.9
No	6	2 (33.3)	4.3–77.7	5 (83.3)	35.9–99.6
**ECOG PS**					
0	14	4 (28.6)	8.4–58.1	12 (85.7)	57.2–98.2
1	19	4 (21.1)	6.1–45.6	13 (68.4)	43.4–87.4
**Smoking status**					
Current/former smoker	31	8 (25.8)	11.9–44.6	23 (74.2)	55.4–88.1
Never smoked	2	0	0–84.2	2 (100)	15.8–100
**Disease stage**					
Limited	6	1 (16.7)	0.4–64.1	6 (100.0)	54.1–100.0
Extensive	27	7 (25.9)	11.1–46.3	19 (70.4)	49.8–86.2
**Response to first-line platinum therapy**					
Platinum-sensitive[a]	17	7 (41.2)	18.4–67.1	15 (88.2)	63.6–98.5
Platinum-resistant[b]	16	1 (6.3)	0.2–30.2	10 (62.5)	35.4–84.8
**Brain metastases**					
Yes	5	1 (20.0)	0.5–71.6	3 (60.0)	14.7–94.7
No	28	7 (25.0)	10.7–44.9	22 (78.6)	59.0–91.7
**Liver metastases**					
Yes	7	1 (14.3)	0.4–57.9	6 (85.7)	42.1–99.6
No	26	7 (26.9)	11.6-47.8	19 (73.1)	52.2–88.4
**Baseline lactate dehydrogenase**					
≤ ULN[c]	21	5 (23.8)	8.2–47.2	16 (76.2)	52.8–91.8
> ULN[c]	12	3 (25.0)	5.5–57.2	9 (75.0)	42.8–94.5
**PD-L1 combined positive score**					
≥ 1	14	5 (35.7)	12.8–64.9	12 (85.7)	57.2–98.2
< 1	16	3 (18.8)	4.0-45.6	11 (68.8)	41.3–89.0
Missing	3	0	0–70.8	2 (66.7)	9.4–99.2
**Prophylactic peg-GCSF administration[d]**					
Yes	13	5 (38.5)	13.9–68.4	10 (76.9)	46.2–95.0
No	20	3 (15.0)	3.2–37.9	15 (75.0)	50.9–91.3

[a]Defined as a progression-free interval ≥90 days after completion of platinum therapy, per case report form.
[b]Defined as a progression-free interval <90 days after completion of platinum therapy, per case report form.
[c]Per laboratory standards.
[d]During cycle 1.

CI, confidence interval; ECOG PS, Eastern Cooperative Oncology Group performance status; ICI, immune checkpoint inhibitor peg-GCSF, pegylated granulocyte colony-stimulating factor; ULN, upper limit of normal.
