# Supplementary material for: Phase II Study of the Liposomal Formulation of Eribulin (E7389-LF) in Combination with Nivolumab: Results from the Small Cell Lung Cancer Cohort
Source: Cancer Res Commun. 2024 Jan 29;4(1):226–35. doi: 10.1158/2767-9764.CRC-23-0313 (PMC10823908; doi:10.1158/2767-9764.CRC-23-0313)
Supplement: Supplemental Table 3 — Supplementary Table 3. Anticancer Therapies Received During Survival Follow-up [file crc-23-0313-s08.pdf]

Supplementary Table 3. Anticancer Therapies Received During Survival Follow-up

**Category**	**Patients (n = 33)**
**Number of anticancer medication regimens during survival follow-up, patient n (%)**	
1	14 (42.4)
2	8 (24.2)
**Subsequent anticancer medications received, patient n (%)**	22 (66.7)
Amrubicin	18 (54.5)
Etoposide	7 (21.2)
Carboplatin	6 (18.2)
Irinotecan	3 (9.1)
Cisplatin	1 (3.0)
Datopotamab deruxtecan	1 (3.0)
Durvalumab	1 (3.0)
Topotecan	1 (3.0)
