# Supplementary material for: Phase II Study of the Liposomal Formulation of Eribulin (E7389-LF) in Combination with Nivolumab: Results from the Small Cell Lung Cancer Cohort
Source: Cancer Res Commun. 2024 Jan 29;4(1):226–35. doi: 10.1158/2767-9764.CRC-23-0313 (PMC10823908; doi:10.1158/2767-9764.CRC-23-0313)
Supplement: Supplemental Table 4 — Supplementary Table 4. Median Change in Biomarkers from Baseline [file crc-23-0313-s09.pdf]

Supplementary Table 4. Median Change in Biomarkers from Baseline

**Biomarker**	**C1D8 (n = 33)**		**C2D1 (n = 30)**		**C2D8 (n = 29)**		**C3D1 (n = 25)**	
	**Med % Δ from Baseline**	**P-value**	**Med % Δ from BL**	**P-value**	**Med % Δ from Baseline**	**P-value**	**Med % Δ from Baseline**	**P-value**
Angiopoietin 2	**23.1**	**<0.001**	8.0	0.184	10.5	0.015	6.9	0.378
BAFF (B cell-activating factor)	**150.7**	**<0.001**	**37.5**	**<0.001**	**155.6**	**<0.001**	**58.9**	**<0.001**
BLC (B lymphocyte chemoattractant)	**44.3**	**<0.001**	13.9	0.048	23.2	0.013	7.1	0.066
Cancer antigen 15 3	−4.5	0.848	0.6	0.144	0.0	0.187	0.0	0.522
CA9 (carbonic anhydrase-9)	**155.3**	**<0.001**	2.6	0.504	**131.6**	**<0.001**	0.0	0.828
6Ckine	1.5	0.760	5.7	0.090	0.7	0.841	6.1	0.037
Collagen IV	**158.2**	**<0.001**	**61.8**	**<0.001**	**128.0**	**<0.001**	**72.0**	**<0.001**
Decorin	**76.9**	**<0.001**	6.3	0.109	**46.2**	**<0.001**	8.3	0.049
EGFR (epidermal growth factor receptor)	0.0	0.546	−2.9	0.568	2.5	0.241	-3.1	0.945
Endoglin	**12.1**	**0.008**	7.4	0.029	**15.4**	**0.011**	6.1	0.072
Fatty acid-binding protein, adipocyte	**145.2**	**<0.001**	**21.3**	**0.001**	**110.7**	**<0.001**	11.8	0.015
Factor VII	5.7	0.039	1.5	0.912	**17.1**	**<0.001**	2.8	0.314
Heparin-binding EGF-like growth factor	**12.4**	**0.016**	−1.7	0.521	7.1	0.056	-8.3	0.525
Hepsin	**7.9**	**0.002**	**13.6**	**0.004**	**18.2**	**<0.001**	14.3	0.025
HER2 (human epidermal growth factor receptor 2)	0	0.695	**12.6**	**0.007**	**15.8**	**0.002**	9.3	0.037
HGFR (hepatocyte growth factor receptor)	**−10.0**	**0.017**	2.4	0.204	2.7	0.825	2.7	0.556
ICAM1 (intercellular adhesion molecule 1)	**31.4**	**<0.001**	**17.7**	**0.001**	**36.6**	**<0.001**	**24.2**	**0.004**
IFNγ (interferon gamma)	**214.3**	**<0.001**	**36.0**	**0.030**	**226.3**	**<0.001**	18.2	0.198
IGFBP1 (insulin-like growth factor-binding protein 1)	**111.3**	**<0.001**	**73.7**	**<0.001**	**130.6**	**<0.001**	**75.4**	**<0.001**
IGFBP2 (insulin-like growth factor-binding protein 2)	**−13.9**	**0.014**	4.7	0.380	−12.6	0.141	-3.8	0.927
IL13 (interleukin 13)	**18.2**	**0.007**	**12.5**	**0.107**	−7.1	0.108	0.0	0.167
IL18 (interleukin 18)	**87.7**	**<0.001**	**15.2**	**0.006**	**99.4**	**<0.001**	**30.5**	**<0.001**
IL18BP (interleukin 18 binding protein)	**63.6**	**<0.001**	**16.5**	**<0.001**	**72.4**	**<0.001**	**25.0**	**<0.001**
Interleukin 1 receptor antagonist	**100.7**	**<0.001**	**35.1**	**<0.001**	**120.0**	**<0.001**	0.0	0.143
IL8 (interleukin 8)	**106.2**	**<0.001**	−3.2	0.205	7.4	0.005	0.0	0.492

Biomarker	C1D8 (n = 33)		C2D1 (n = 30)		C2D8 (n = 29)		C3D1 (n = 25)	
	Med % Δ from Baseline	P-value	Med % Δ from BL	P-value	Med % Δ from Baseline	P-value	Med % Δ from Baseline	P-value
IP10 (interferon gamma-induced protein 10, CXCL10)	**110.6**	**<0.001**	**10.5**	**0.098**	**69.4**	**<0.001**	9.3	0.064
ITAC (interferon-inducible T-cell alpha chemoattractant, CXCL11)	**57.6**	**<0.001**	**21.4**	**0.002**	**113.6**	**<0.001**	**63.2**	**0.001**
Kallikrein 5	**20.0**	**0.008**	−2.0	0.704	9.5	0.103	0.0	0.263
Kallikrein 7	−3.3	0.715	**−12.8**	**0.011**	−2.9	0.894	-7.7	0.244
MCP1 (monocyte chemotactic protein 1)	**158.2**	**<0.001**	−12.1	0.592	**102.9**	**<0.001**	-17.0	0.226
MIF (macrophage migration inhibitory Factor)	9.5	0.240	−33.5	0.154	0.0	0.894	-31.8	0.021
MIG (monokine induced by gamma interferon, CXCL9)	**18.0**	**0.006**	10.8	0.162	**36.7**	**0.027**	-4.9	0.154
MIP1β (macrophage inflammatory protein-1 beta)	**119.7**	**<0.001**	0.3	0.924	**100.3**	**<0.001**	2.2	0.685
MIP3β (macrophage inflammatory protein-3 beta)	**52.9**	**<0.001**	4.3	0.107	**22.6**	**<0.001**	20.2	0.066
MMP3 (matrix metallopeptidase 3)	**49.1**	**<0.001**	**19.4**	**<0.001**	**55.6**	**<0.001**	13.3	0.021
MMP9 (matrix metallopeptidase 9)	−13.9	0.227	5.1	0.119	0.0	0.739	-9.1	0.803
PECAM1 (platelet endothelial cell adhesion molecule 1)	**10.9**	**<0.001**	**13.0**	**<0.001**	**23.3**	**<0.001**	**18.6**	**<0.001**
Prostasin	4.0	0.425	−5.2	0.912	**16.5**	**0.002**	7.1	0.064
SCF (stem cell factor)	6.2	0.079	0.0	0.364	15.2	0.041	15.2	0.232
SDF1 (stromal cell-derived factor 1, CXCL12)	**20.9**	**<0.001**	6.3	0.154	**28.3**	**<0.001**	2.2	0.523
SPD (pulmonary surfactant-associated protein)	**−28.0**	**<0.001**	−9.1	0.385	**−23.7**	**<0.001**	6.4	0.244
TG (thyroglobulin)	**31.3**	**<0.001**	0	0.678	0	0.342	-15.6	0.798
TIE2 (tyrosine kinase immunoglobulin and epidermal growth factor homology domains 2, TEK)	**54.5**	**<0.001**	**20.0**	**<0.001**	**60.0**	**<0.001**	**23.1**	**<0.001**
TNC (tenascin-C)	**318.3**	**<0.001**	**76.3**	**<0.001**	**435.7**	**<0.001**	**102.6**	**<0.001**

Biomarker	C1D8 (n = 33)		C2D1 (n = 30)		C2D8 (n = 29)		C3D1 (n = 25)	
	**Med % Δ from Baseline**	**P-value**	**Med % Δ from BL**	**P-value**	**Med % Δ from Baseline**	**P-value**	**Med % Δ from Baseline**	**P-value**
UPAR (urokinase-type plasminogen activator receptor)	**31.7**	**<0.001**	**10.9**	**<0.001**	**40.8**	**<0.001**	**31.3**	**<0.001**
VEGF (vascular endothelial growth factor)	10.5	0.171	−13.4	0.102	−0.9	0.708	-13.6	0.014
VEGFD (vascular endothelial growth factor D)	**5.9**	**0.016**	2.9	0.297	2.1	0.162	-0.6	0.435
VEGFR2 (vascular endothelial growth factor receptor 2)	**−13.6**	**<0.001**	−3.0	0.580	−2.9	1.000	0.0	0.978
VEGFR3 (vascular endothelial growth factor receptor 3)	6.1	0.285	2.7	0.154	**12.8**	**0.005**	16.4	0.017

P-values shown are of Wilcoxon signed-rank tests; **bolded** values were significant after false-discovery-rate adjustment.

C#D#, cycle # day #; CCL, C-C motif chemokine ligand; CXCL, C-X-C motif chemokine ligand; TEK, TEK receptor tyrosine kinase.
