# Supplementary material for: Phase II Study of the Liposomal Formulation of Eribulin (E7389-LF) in Combination with Nivolumab: Results from the Small Cell Lung Cancer Cohort
Source: Cancer Res Commun. 2024 Jan 29;4(1):226–35. doi: 10.1158/2767-9764.CRC-23-0313 (PMC10823908; doi:10.1158/2767-9764.CRC-23-0313)
Supplement: Supplemental Table 5 — Supplementary Table 5. Median Change in Biomarkers from Baseline Over Time by Tumor Response [file crc-23-0313-s10.pdf]

Supplementary Table 5. Median Change in Biomarkers from Baseline Over Time by Tumor Response

	**C1D8**			**C2D1**			**C3D1**			**C4D1**		
**CR or PR**	**Yes (n = 8)**	**No (n = 25)**		**Yes (n = 8)**	**No (n = 22)**		**Yes (n = 8)**	**No (n = 17)**		**Yes (n = 8)**	**No (n = 16)**	
**Biomarker**	**Med % Δ from BL**	**Med % Δ from BL**	**P-value**	**Med % Δ from BL**	**Med % Δ from BL**	**P-value**	**Med % Δ from BL**	**Med % Δ from BL**	**P-value**	**Med % Δ from BL**	**Med % Δ from BL**	**P-value**
Collagen IV	171.6	154.6	0.488	60.3	61.8	0.907	81.8	30.6	0.256	112.5	58.3	0.257
Endoglin	14.9	12.1	0.834	6.2	7.4	0.656	10.2	4.3	0.705	16.8	−8.2	0.284
ICAM1 (intercellular adhesion molecule 1)	28.7	31.4	0.785	10.1	21.5	0.622	31.9	12.5	0.793	18.4	0.9	0.783
IFNγ (interferon gamma)	253.7	187.0	0.542	3.4	40.5	0.527	−8.6	18.2	0.432	15.4	−1.0	0.878
IP10 (interferon gamma-induced protein 10, CXCL10)	81.8	135.4	0.413	−10.8	19.1	0.313	13.6	9.3	0.705	5.5	−19.2	0.691
ITAC (interferon-inducible T-cell alpha chemoattractant, CXCL11)	136.7	53.1	0.449	45.4	4.7	0.225	80.0	37.1	0.815	25.0	0	0.292
MIG (monokine induced by gamma interferon, CXCL9)	18.4	18.0	0.515	−25.5	14.7	0.360	−28.1	15.1	0.154	−16.5	17.0	0.168
PECAM1 (platelet endothelial cell adhesion molecule 1)	13.2	10.9	0.571	9.9	13.3	0.981	24.7	8.8	0.580	**23.3**	**8.5**	**0.062**
TIE2 (tyrosine kinase immunoglobulin and epidermal growth factor homology domains 2, TEK)	55.4	53.8	0.966	22.0	20.0	0.743	**45.1**	**16.7**	**0.097**	**45.3**	**21.5**	**0.018**
VEGFR3 (vascular endothelial growth factor receptor 3)	3.0	7.7	0.556	5.4	2.1	0.870	9.0	16.4	0.749	−1.5	4.4	0.713

P-values shown are of Wilcoxon rank-sum tests; they measure the difference in biomarker changes between those who had a CR/PR, and those who did not. Bolded values represent a p-value of <0.1.

BL, baseline; C#D#, cycle # day #; CCL, C-C motif chemokine ligand; CR, complete response; CXCL, C-X-C motif chemokine ligand; Ig, immunoglobulin; PR, partial response; TEK, TEK receptor tyrosine kinase.
